# In Vitro Reduced Susceptibility to Pentavalent Antimonials of a *Leishmania infantum* Isolate from a Human Cutaneous Leishmaniasis Case in Central Italy

**DOI:** 10.3390/microorganisms9061147

**Published:** 2021-05-26

**Authors:** Aurora Diotallevi, Gloria Buffi, Giovanni Corbelli, Marcello Ceccarelli, Margherita Ortalli, Stefania Varani, Mauro Magnani, Luca Galluzzi

**Affiliations:** 1 Department of Biomolecular Sciences, University of Urbino “Carlo Bo”, 61029 Urbino, Italy; aurora.diotallevi@uniurb.it (A.D.); g.buffi@campus.uniurb.it (G.B.); m.ceccarelli3@campus.uniurb.it (M.C.); mauro.magnani@uniurb.it (M.M.); 2 Unit of Infectious Diseases, Marche Nord Hospital, 61122 Pesaro, Italy; giovanni.corbelli@ospedalimarchenord.it; 3 Unit of Microbiology, IRCCS Polyclinic S.Orsola-Malpighi, 40138 Bologna, Italy; margherita.ortalli@gmail.com (M.O.); stefania.varani@unibo.it (S.V.); 4 Department of Experimental, Diagnostic and Specialty Medicine, Alma Mater Studiorum University of Bologna, 40126 Bologna, Italy

**Keywords:** *Leishmania infantum*, antimonials, resistance, cutaneous leishmaniasis

## Abstract

Cutaneous leishmaniasis (CL) caused by *Leishmania* (*Leishmania*) *infantum* is endemic in the Mediterranean basin. Here we report an autochthonous case of CL in a patient living in central Italy with an unsatisfactory response to treatment with intralesional Meglumine Antimoniate and in vitro demonstration of reduced susceptibility to SbIII. Parasitological diagnosis was first achieved by histopathology on tissue biopsy and the patient was treated with a local infiltration of Meglumine Antimoniate. Since the clinical response at 12 weeks from the treatment’s onset was deemed unsatisfactory, two further skin biopsies were taken for histopathological examination, DNA extraction and parasite isolation. *L. (L.) infantum* was identified by molecular typing. The low susceptibility to Meglumine Antimoniate was confirmed in vitro: the promastigotes from the patient strain showed significantly lower susceptibility to SbIII (the active trivalent form of antimonial) compared to the reference strain MHOM/TN/80/IPT1. The patient underwent a new treatment course with intravenous liposomal Amphotericin B, reaching complete healing of the lesion. Additional studies are needed to confirm the epidemiological and clinical relevance of reduced susceptibility to SbIII of human *L.* (*L*.) *infantum* isolate in Italy.

## 1. Introduction

Leishmaniases are parasitic diseases transmitted by sandflies showing heterogeneous clinical manifestation, depending on the *Leishmania* species and host health status. About 20 species of *Leishmania*, mainly belonging to the subgenera *Leishmania* and *Viannia*, can parasitize humans. A comprehensive updated taxonomy of trypanosomatidae, including the genus *Leishmania*, has been recently published [1]. Clinical manifestations range from cutaneous lesions (cutaneous leishmaniasis, CL) to severe systemic multiorgan disease (visceral leishmaniasis, VL). In the Old World (i.e., southern Europe, the Middle East, Asia, and Africa) the etiological agents of CL are *Leishmania* (*Leishmania*) species, such as *L.* (*L.*) *donovani, L.* (*L.*) *infantum, L.* (*L.*) *major, L.* (*L.*) *aethiopica,* and *L.* (*L.*) *tropica* [2]. The lesions generally occur on skin portions easily accessible to sand flies, such as the face or limbs. The CL lesions can manifest as a single nodular or ulcerative lesion at the site of parasite inoculation, called localized cutaneous leishmaniasis (LCL) or as multiple lesions caused by infection propagation, named diffuse cutaneous leishmaniasis (DCL) [3]. In the Mediterranean basin, CL often manifests as a single, painless lesion caused by *L.* (*L.*) *infantum* [4], which represents the etiological agent of CL and VL in humans, as well as canine leishmaniasis (CanL).

In the Old World, intralesional pentavalent antimony compounds (i.e., sodium stibogluconate and Meglumine Antimoniate) are among the first choice for the treatment of uncomplicated CL, while liposomal amphotericin B or other systemic treatments can be used for complicated CL or for immunosuppressed patients [5]. Antimonials are used also in the standard therapy for CanL, either alone, or in combination with allopurinol [6]. Notably, treatment failure due to resistance to antimonials has been described in different studies, mostly in the treatment of *L.* (*L.*) *donovani* VL in the Indian subcontinent [7], and rarely in the Mediterranean region [8], where treatment efficiency exceeding 95% in HIV-negative individuals has been reported [9].

In recent years, new evidence of CL cases in north-eastern Italy has been documented [10]. However, to the best of our knowledge, human CL cases not responding to antimonials have never been notified in Italy so far. Here, we report an autochthonous CL case due to *L.* (*L.*) *infantum* in central Italy, with unsatisfactory response to treatment with intralesional pentavalent antimony compounds.

## 2. Description of the Case 

A 61-year-old male was referred from the San Salvatore–Muraglia hospital (Pesaro, Italy), in May 2019 as a suspected case of CL, with a skin lesion on the dorsal left forearm with diameter of 5 × 3 cm. The patient reported not having traveled abroad in the previous years. Physical examination did not reveal any clinical sign of pathology, except for the abovementioned lesion. The lesion was biopsied by a dermatologist in aseptic conditions, with histological report of skin characterized by intense chronic limphoplasmocytoid inflammation and presence of histiocytes containing several *Leishmania* amastigotes. The ensuing pathological diagnosis was cutaneous leishmaniasis of the left forearm. Laboratory results showed 5.81 × 10^6^ erythrocytes, Hb 17.1 g/dL, MCV 88, normal white blood cells, and platelet count and biochemical parameters within normal range. Renal and liver function were also normal, and no autoantibodies were detected. The patient had just been tested for polyglobulia by a hematologist, and he was in clinical follow-up. No evidence of BCR-ABL or Jak2 mutations was reported.

**Figure 1:**
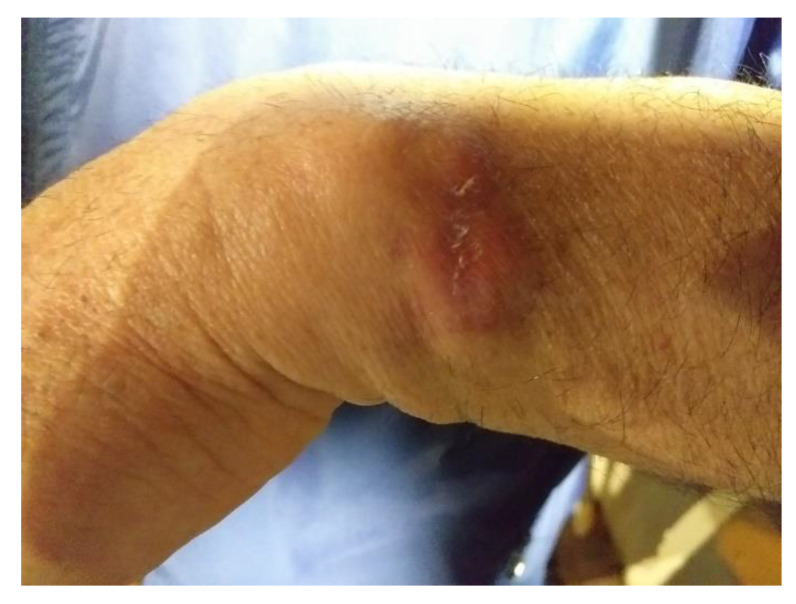
Photograph of the dorsal forearm skin lesion on the patient with cutaneous leishmaniasis, twelve weeks after the beginning of treatment.

The patient was treated with a local infiltration of Meglumine Antimoniate (Glucantime^®^ vials 1.5 g/5 mL—1 vial every week for a total of 5 weeks) in the skin lesion and along its margins. Twelve weeks after the beginning of therapy the clinical result was not deemed satisfactory by the managing clinicians (Figure 1). 

**Figure 2:**
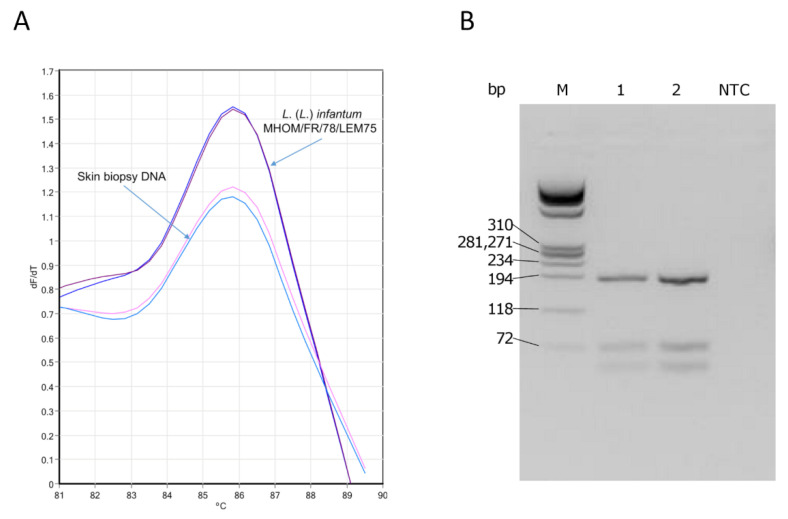
Molecular identification and characterization of the parasite from the skin biopsy. (**A**) Melting analysis of qPCR-ML amplicons. A region of kinetoplast DNA (kDNA) was amplified by a qPCR assay using primer pairs MLF/MLR as described previously [13]. Briefly, PCR reactions were carried out in duplicate, in 25 µL volume containing 1 µL template DNA (corresponding to 140 ng DNA) and 24 µL SYBR green PCR master mix (Diatheva srl) with 200 nM of each primer, using a Rotor-Gene 6000 instrument (Corbett life science). The amplification conditions were: 94 °C for 10 min; followed by 45 cycles at 94 °C for 20 s, 60 °C for 20 s, and 72 °C for 20 s. At the end of the run, a melting curve analysis was performed from 82 °C to 90 °C. As positive control, DNA from *L*. (*L*.) *infantum* MHOM/FR/78/LEM75 was used. Melting temperatures of PCR products were overlapping (85.8 °C) indicating that parasites were from *Leishmania* (*Leishmania*) subgenus. (**B**) ITS1-PCR RFLP analysis. ITS1 region was amplified by PCR as described previously [16] using primers LITSR 5′-CTGGATCATTTTCCGATG-3′ and L5.8 S 5′-TGATACCACTTATCGCACTT-3′. ITS1 PCR products obtained from skin biopsy (1) and *L. (L.) infantum* MHOM/FR/78/LEM75 (2) were digested with 10 U HaeIII enzyme (Thermo Fisher Scientific) at 37 °C for 3 h and visualized on a 3.5% high-resolution MetaPhor (Cambrex) agarose gel stained with GelRed (Biotium). NTC, no template control; M, DNA marker 9.

**Figure 3:**
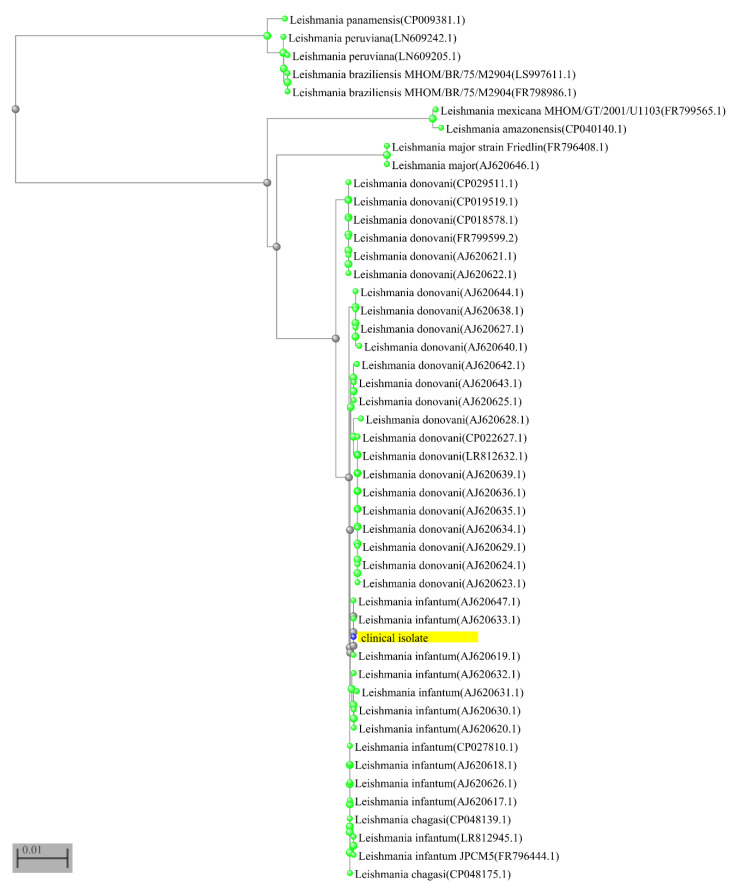
The partial sequence of glucose-6-phosphate isomerase gene (1335 bp) was obtained from isolated parasites in the context of a multilocus sequence typing approach by a customized sequencing panel designed with Ion AmpliSeq™ designer (Thermo-Fisher-Scientific). This panel included 7 primer pairs specific for the glucose-6-phosphate isomerase gene. The library was prepared using Ion AmpliSeq™ library kit plus (Thermo-Fisher-Scientific) following manufacturer’s instructions. The library sequencing was performed using the Ion Torrent S5 instrument (Thermo-Fisher-Scientific) and the reads were mapped to *L. infantum* JPCM5 genome (LinJ.12 291520-292854) using Torrent Browser. The consensus sequence was analyzed by BLASTN against *Leishmania* sequences. The results with 100% coverage were selected. A distance tree of pairwise comparisons was visualized by BLAST tree view, using Neighbor Joining algorithm [17]. The sequence of the clinical isolate is highlighted.

After 12 weeks from the beginning of treatment, the patient underwent a second skin biopsy. Maintaining aseptic conditions and after the expression patient’s informed consent, two 5 mm diameter biopsies were collected from the nodular lesion. One biopsy was fixed in 4% formalin and sent for microscopic analysis which showed persistence of histiocytes containing *Leishmania* amastigotes. The second biopsy was collected in 5 mL sterile Tobie medium, disrupted by pipetting and divided into two aliquots: one was used for parasite isolation as described previously [11,12] and the other for DNA extraction. The DNA was extracted with the DNeasy Blood & Tissue kit (Qiagen) and amplified by a real-time PCR assay (qPCR-ML) as previously described [13,14,15]. The qPCR-ML assay gave positive amplification results indicating the presence of *Leishmania* spp DNA in the skin sample. Melting analysis [13] allowed amplicons to be assigned to *Leishmania* (*Leishmania*) subgenus (Figure 2A). The ITS1-PCR RFLP analysis, performed as described by Schönian et al. [16], enabled the identification of *L.* (*L*.) *infantum* species (Figure 2B). The species identification was confirmed by partial sequencing of glucose-6-phosphate isomerase gene and successive alignment against *Leishmania* sequences using the BLASTN algorithm, followed by construction of the phylogenetic tree (Figure 3).

**Figure 4:**
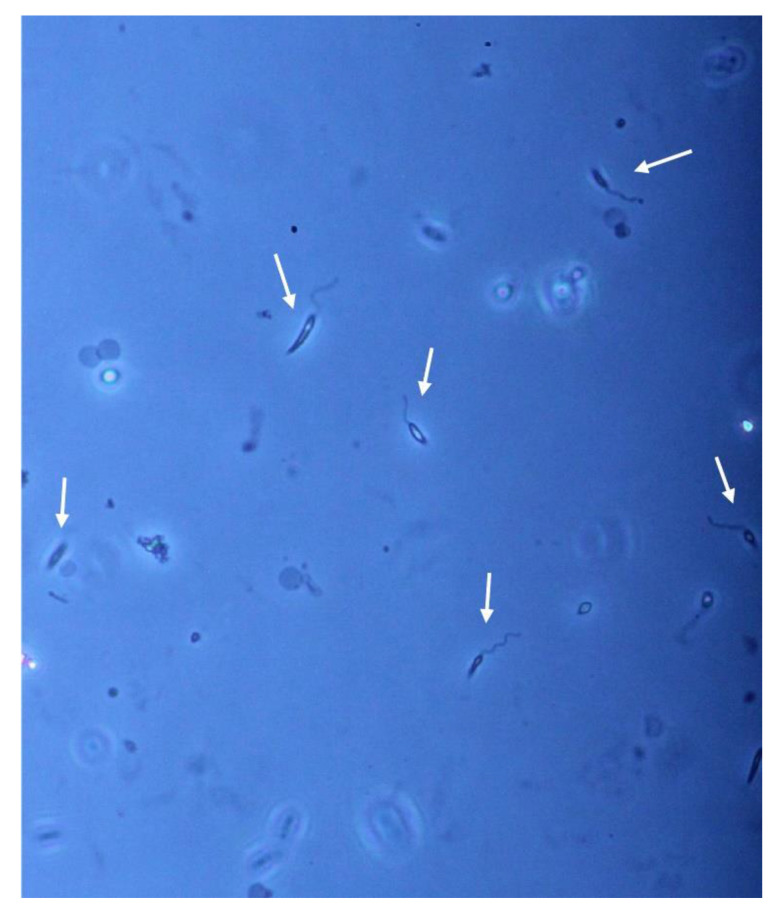
Phase-contrast microscope examination of parasites isolated from the skin biopsy. Individual promastigotes are indicated by arrows (20× magnification).

Regarding parasite isolation, after 7 days of culture, the liquid phase of Evans’ modified Tobie’s medium (EMTM) presented numerous motile promastigotes, confirming the presence of viable parasites in the bioptic sample (Figure 4). 

**Figure 5:**
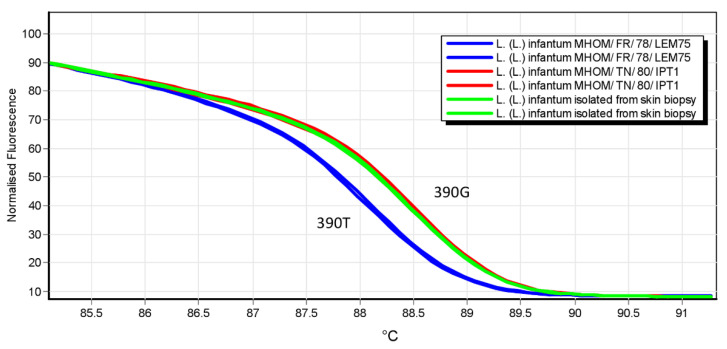
Genotyping of *L. (L.) infantum* clinical isolate through HRM analysis. The malic enzyme gene sequence encompassing the polymorphism 390 T/G was amplified by qPCR-MEint in 25 µL volume containing SYBR green PCR master mix (Diatheva srl) with 200 nM of primers forward (5′-TCAGAACCTTCGCAAGACGA-3′) and reverse (5′-CACTTGCCGATGCTGATGC-3′), using a Rotor-Gene 6000 instrument (Corbett life science) [18]. The amplification conditions were: 94 °C for 10 min; followed by 45 cycles at 94 °C for 20 s, 60 °C for 20 s, and 72 °C for 20 s. After amplification, high-resolution melting (HRM) analysis was performed over the range 77–95 °C, rising by 0.1 °C/s and waiting for 2 s at each temperature. Raw HRM curves were normalized by the Rotorgene 6000 v.1.7 software. Controls for genotypes 390 T and 390 G were *L. (L.) infantum* MHOM/FR/78/LEM75 and *L. (L.) infantum* MHOM/TN/80/IPT1, respectively.

Furthermore, the isolate was genotyped by analyzing the nucleotide polymorphism 390 T > G in the malic enzyme gene by High Resolution Melt (HRM) analysis, as described previously [18]. The HRM analysis showed a 390 G genotype (Figure 5), which is not associated with the zymodemes MON-1 (the most common in the Mediterranean basin), MON-72, 201 [18]. 

To determine whether unresponsiveness to Glucantime^®^ was due to the presence of resistant parasites, the IC_50_ of SbIII (the active trivalent antimonial form) was determined in the promastigotes isolated from the lesion. Briefly, late log/stationary phase promastigotes were resuspended in complete RPMI-PY medium [19] at a density of 2.5 × 10^6^ parasites/mL in 96-well plates (100 μL/well). The promastigotes were treated with potassium antimonyl tartrate trihydrate (SbIII) (Sigma-Aldrich) at concentrations of 1, 5, 25, 125, 625 μM for 72 h at 26 °C. The reference strain *L. (L.) infantum* MHOM/TN/80/IPT1 was included as control. Moreover, the promastigotes were also treated with Miltefosine (Sigma–Aldrich). Each condition was repeated sixfold. To evaluate the promastigote viability, the CellTiter 96 H Aqueous Non-Radioactive Cell Proliferation Assay (Promega) was carried out. The parasites derived from the unresponsive patient were significantly less susceptible to SbIII than the reference strain parasites (*p <* 0.01, unpaired t-test with Welch’s correction) (Table 1). On the contrary, promastigotes from the clinical isolate appeared more sensitive to Miltefosine compared to the reference strain. 

Due to the unsatisfactory clinical response to pentavalent antimony compounds and the in vitro demonstration of low susceptibility to antimonials of the infecting parasitic strain, a new therapy with intravenous liposomal Amphotericin B was administered at the dose of 3 mg/Kg in daily doses for five days, with two further doses at an interval of two weeks. Liposomal Amphotericin B treatment was effective, with healing of the lesion after two months and no major side-effect was reported during the follow-up. The patient did not return with any relapse in the following 6 months.

## 3. Discussion

A high circulation of *Leishmania* strains causing cutaneous leishmaniasis has been recently observed in northeastern Italy [10]. In the index case, a lack of travel history, the molecular identification of *L. (L.) infantum* and the fact that the patient resided in the Marche region (central Italy) where *L. (L.) infantum* is endemic, strongly suggest an autochthonous origin of the infection. 

The diagnosis of CL was initially performed by histology with the detection of leishmanial amastigotes in tissue sections. However, due to treatment unresponsiveness, a second bioptic sample was taken and histological diagnosis was repeated, followed by molecular diagnosis and parasite isolation and characterization. 

Treatment failure in leishmaniasis can be caused by drug resistance of the infecting parasite, host factors such as immunity and nutritional status, individual variation in pharmacokinetics, or other drug-related responses, or whether the parasite resides in tissues accessible to drugs [7]. Antimonials are often the first choice in the treatment of CL and they can be administered intralesionally as well as systemically [20]. In Old World CL, intralesional antimonials have shown >90% cure rates but most of the data are related to *L. (L.) major* infections. [20]. In fact, treatment data are scarce for *L. (L.) infantum* CL lesions [21]. To confirm the first diagnosis and to investigate the lack of response to treatment in the index case, the parasitic strain isolated from the lesion was further characterized with molecular tools and its susceptibility to antimonials was evaluated in vitro. Pentavalent antimonials (SbV) such as Meglumine Antimoniate need to be reduced to a trivalent antimonial (SbIII) in order to be active. Since promastigotes cannot reduce SbV to SbIII, the susceptibility test was directly performed with SbIII, the active trivalent antimonial form [22]. 

The *L*. (*L.*) *infantum* clinical isolate showed significantly lower susceptibility to antimonials than the reference strain (MHOM/TN/80/IPT1), which is itself a VL strain less susceptible to antimonials with respect to CL causing species [23]. At the same time, the clinical isolate exhibited a higher sensitivity to Miltefosine when compared to the reference strain. 

Treatment of CanL with Glucantime^®^ is a common practice in many Mediterranean countries, where repeated treatments of dogs have been shown to produce a reservoir of *L*. (*L.*) *infantum* parasites with a decreased sensitivity to antimonials [24,25]. Nevertheless, the clinical isolate of our index case was genotyped as a strain not related to zymodemes MON-1, 72, 201 (polymorphism 390 G in malic enzyme), which include the most common zymodeme circulating in dogs in the Mediterranean basin (MON-1). Therefore, it is unlikely that the strain has canine origin. Since the lower susceptibility to SbIII could not be explained with canine origin of the strain, further investigation on genes involved in inducing antimony-resistant parasites (e.g., AQP1, MRPA, γ-GCS, TR, TDR1) will be needed.

According to the European guidelines [5], CL lesions > 4 cm should be treated with systemic therapy. However, Since the patient presented a single lesion that was slightly above this index, local therapy with Glucantime^®^ was attempted to avoid the toxic effects of the systemic therapy. Nonetheless, after the intralesional therapy, according to the clinician’s judgment, a satisfactory clinical response was not achieved. Subsequent investigations revealed low susceptibility to SbIII. Therefore, the incomplete response to treatment could be due to both an insufficient therapeutic dose and the low susceptibility of the parasite.

In the Old World, Glucantime^®^ failure due to parasite resistance has been reported mostly in *L.* (*L.*) *donovani* strains circulating in the Indian subcontinent. The *L.* (*L.*) *infantum* CL case described here showed decreased susceptibility to Meglumine Antimoniate. Due to the scarcity of data regarding treatment of *L. (L.) infantum* CL lesions, it is possible that some similar cases may not have been reported. Additional studies are needed to confirm the epidemiological and clinical relevance of these findings. 

## Figures and Tables

**Table 1:** In vitro susceptibility tests.

Treatment	*L. (L.) infantum* Clinical Isolate IC_50_ (μM)	*L. (L.) infantum* MHOM/TN/80/IPT1 IC_50_ (μM)	Unpaired *t*-Test with Welch’s Correction *p*-Value
Potassium antimonyl tartrate trihydrate (SbIII)	27.06 ± 2.70	13.22 ± 2.11	<0.01
Miltefosine	1.31 ± 0.14	4.27 ± 0.40	<0.01

## Data Availability

Data sharing not applicable. All presented data are included in the manuscript.
